# How to Avoid Taking Cold

**Published:** 1894-01

**Authors:** Chas. A. Hough


### SELECTIONS.

### How to Avoid Taking Cold.

BY CHAS. A. HOUGH, M.D.

To those who " take cold easily " the usual caution against draughts and damp feet is inadequate. Such persons seldom suffer from a known imprudence. Little keyhole draughts and " trifling " exposures, like little sins, which no remorse attends or cures, are most dangerous to delicate persons. These slight causes of disease, which are harmless to robust persons, can never be entirely avoided, and it is only by increasing the body's power of resisting such influences, that this unfortunate susceptibility to colds may be cured. This power of resistance may be cultivated. Autumn is the most favorable season for such treatment and the cure may be wrought at home and without expense.

The skin is much more than a mere covering to the body. It might be said to represent in its structure, and to supplement in functions almost every organ of the body. The blushing cheek tells how intimate is the relation of blood supply to nervous impression. So richly supplied with blood vessels that it may at one time contain a large proportion of the entire blood; so intimately related to the vital processes that a burn of even slight severity extending over more than three-fifths of the body is generally fatal, and so delicately sensitive that differences in weight of seven to fifteen grains, and variations in temperature of but one-half degree are perceptible, we need not wonder that through our skin we are profoundly influenced by our surroundings.

The influence of cold upon the skin causes a temporary blanching of the surface; the minute blood vessels contract; the blood recedes and accumulates in deeper and more protected structures. That which immediately follows this first chilling is wherein the person of robust health and the one who "takes cold easily" differ so widely. Immediately following the blanching should be a return flow of warm blood to the exposed part, the skin becoming rosy, warm and doubly fortified against chill. The delightful glow which follows bathing a part of the body in cold water is an example of what should follow exposure. Reaction often follows so quickly that stepping from the house into cold wind or rain appears to call an immediate glow to the face, and warmth to the entire body. Persons who are endowed with this power of rapid reaction enjoy boisterous weather and have little fear of evil consequences from an ordinary exposure.

Vastly different is the lot of their unfortunate fellows who take cold easily. Even a slight chilling of the skin produces effects which may ultimately be serious. The circulation, usually sluggish and insufficient in quantity, is profoundly disturbed. Blanching of the exposed portion of the body is pronounced. The skin becomes relaxed, white, moist and cold, or the condition called "goose-skin" may be present. This local condition persists possibly for hours. The nervous system is profoundly impressed; general chilliness, indisposition, both physical and mental, frontal headache, aching of the limbs, general soreness —in short, what physicians call general malaise, or "ill ease," indicate an imminent illness. All these results attend because there is failure to react at the point of exposure.

Now, it is by cultivating the power of reaction that delicate persons may lessen their liability to colds. Multiplying wraps, increasing the weight of clothing ordinarily worn, and hermetically sealing and overheating the living-rooms are all wrong.

Prompt reaction presupposes pure blood and plenty of it circulating in a healthy skin. Pure blood can only be made from proper food—not medicine—assimilated during exercise, either active or passive in pure air not too warm. A healthy skin is, first, a clean skin, one from which all the organic debris has been removed by thorough washing. Too frequent bathing consists in moistening the greasy impurities and then distributing them evenly over the surface—much as we polish a shoe. So long as the moistened hand will rub up any thing from the skin it is not clean.

Next, the skin should be elastic, firm-textured and muscular. Every little hair follicle, each of the myriad glands and all the minute blood vessels with which the skin is so abundantly supplied are surrounded by tiny muscles which have an important role in the processes of life. Unless these muscles be strong, active, quick to respond to demands made upon them by their accompanying nerves, the skin is sluggish and reaction can not be prompt and efficient. These, in common with all muscles, require exercise to keep them in proper tone.

Such a skin as I have attempted to picture may be cultivated, just as quickness of perception, and muscular strength and dexterity in other parts come by exercise.

This may strike one as a novel proposition, but it is absolutely true that the most efficacious means of cultivating immunity against colds is in exercising the skin and habituating it to those changes of temperature and humidity which no one can hope entirely to avoid.

If the reader will give but one month's trial to the plan of treatment I am proposing further argument will be unnecessary.

First, keep your skin clean by frequent, thorough, energetic bathing, followed by much friction. If you are in reach of a competent masseur employ massage occasionally, until your skin acquires elasticity, and becomes hardened to rather harsh usage.

Immediately upon rising move leisurely about the chamber for a few minutes, day by day increasing the exposure of the body, until soon you can take an air bath of five or ten minutes' duration without discomfort. This exposure should always be followed by brisk rubbing before dressing. Soon you may venture to dampen the entire body, by rubbing with the hand moistened in water which has stood exposed over night and is nearly the temperature of the room. Next, use a sponge slightly moistened; then one which is not so dry. Soon you will be taking with impunity and enjoying a sponge bath, which may become more prolonged and more beneficial as the skin becomes habituated to it.

But do not forget that these baths are to be followed, in all cases, by brisk and prolonged rubbing and kneading of the skin, and are not to take the place of the thorough cleansing bath taken at some other time of the day.

These morning baths are merely skin gymnastics, beneficial in that they harden it and increase its power of reaction. As the weather becomes colder with advancing autumn, gradually the morning temperature of your room and the water which you use for your ablutions become lower, and when you are habituated to them, you may venture to open the windows a little on the warmer mornings, and expose the nude body to a slight draught.

During the night the mucous membrane should be hardened by leaving the chamber windows open, to the extent they should have been during the summer, guarding only against draughts or exceptional falls of temperature.

During the day remain out of doors as much as the weather will permit, and resist the impulse to put on unduly heavy clothing.

I would not be understood as deprecating ordinary prudence at any time. Keep the feet dry and warm, and the body dry at all times; avoid violent changes and the long continued cooling of a single portion of the body; remember that the draught through a two-inch aperture, if it strikes the person, is more dangerous than the wind through an entirely open window;

I believe that often the Thanksgiving dinner is as active in causing a cold as are "chill November's surly blasts" outside ; persevere in the course of training laid down, and you will find that what must be endured may be largely curtailed by fortifying against it.

